# A rust fungal effector binds plant DNA and modulates transcription

**DOI:** 10.1038/s41598-018-32825-0

**Published:** 2018-10-03

**Authors:** Md Bulbul Ahmed, Karen Cristine Gonçalves dos Santos, Ingrid Benerice Sanchez, Benjamin Petre, Cécile Lorrain, Mélodie B. Plourde, Sébastien Duplessis, Isabel Desgagné-Penix, Hugo Germain

**Affiliations:** 1 Department of Chemistry, Biochemistry and Physics, Université du Québec à Trois-Rivières (UQTR), Trois-Rivières, QC G9A 5H7 Canada; 2 Groupe de recherche en biologie végétale, UQTR, Trois-Rivières, QC G9A 5H7 Canada; 3 Department of Biotechnology and Engineering in Chemistry, Instituto Tecnológico y de Estudios Superiores de Monterrey, Campus Estado de México (ITESM CEM), Margarita Maza de Juárez, 52926 Cd López Mateos, Mexico; 4 The Sainsbury Laboratory, Norwich Research Park, Norwich, NR4 7UH UK; 5 INRA, UMR 1136 Interactions Arbres/Microorganismes, INRA/Université de Lorraine, Centre INRA Grand Est - Nancy, 54280 Champenoux, France; 6 Université de Lorraine, UMR 1136 Interactions Arbres/Microorganismes, INRA/Université de Lorraine, Faculté des Sciences et Technologies - Campus Aiguillettes, BP 70239–54506 Vandoeuvre-lès-Nancy, France

## Abstract

The basidiomycete *Melampsora larici-populina* causes poplar rust disease by invading leaf tissues and secreting effector proteins through specialized infection structures known as haustoria. The mechanisms by which rust effectors promote pathogen virulence are poorly understood. The present study characterized Mlp124478, a candidate effector of *M. larici-populina*. We used the models *Arabidopsis thaliana* and *Nicotiana benthamiana* to investigate the function of Mlp124478 in plant cells. We established that Mlp124478 accumulates in the nucleus and nucleolus, however its nucleolar accumulation is not required to promote growth of the oomycete pathogen *Hyaloperonospora arabidopsidis*. Stable constitutive expression of *Mlp124478* in *A. thaliana* repressed the expression of genes involved in immune responses, and also altered leaf morphology by increasing the waviness of rosette leaves. Chip-PCR experiments showed that Mlp124478 associats'e with the TGA1a-binding DNA sequence. Our results suggest that Mlp124478 exerts a virulence activity and binds the TGA1a promoter to suppress genes induced in response to pathogen infection.

## Introduction

Plant pathogens secrete molecules, known as effectors, into host tissues to promote parasitic growth. Effectors target various host cell compartments and interact with molecules, such as proteins and DNA, to modulate their location, stability and function^1–4^. Nowadays, molecular plant pathologists employ effectors as probes to identify and understand the plant processes targeted by pathogens and exploit this insight to develop resistant crops. Genomic approaches coupled with heterologous expression studies in *Arabidopsis thaliana* and *Nicotiana benthamiana* are commonly undertaken to decipher the mechanisms by which effectors promote pathogen virulence^5–9^.

Many effectors interfere with transcription to alter plant immune responses^10–12^. For instance, bacterial transcription activator-like effectors (TAL) function as transcription factors and alter host gene expression levels, which may result in substantial influence on host phenotypes^13,14^. The oomycete *Hyaloperonospora arabidopsidis*, a filamentous obligate biotrophic pathogen, has effectors that target the nucleus. One of them, HaRxL44, accumulates in the nucleus and interacts with the Mediator complex MED19a, inducing its proteasome-mediated degradation. This, in turn, leads to transcriptional changes resembling jasmonic acid and ethylene induction with repressed salicylic acid signaling enhancing susceptibility to biotrophs^15^. Similarly, global expression profiling of the fungal biotroph *Ustilago maydis*-maize interaction demonstrated early induction of the defense response genes which are later quenched^16^, indicating that host transcriptional reprogramming is a conserved mechanism amongst biotrophs.

Rust fungi (order *Pucciniales*) are notorious plant pathogens and are among the most studied obligate biotrophic fungal pathogens^17,18^. *Melampsora larici-populina* causes poplar leaf rust disease, which threatens poplar plantations worldwide^19^. Genome analysis of *M. larici-populina* has predicted 1,184 small secreted proteins (SSPs)^20^. Several features, such as expression in poplar leaves during infection, homology to other known rust effectors, signature of positive selection, specificity to Pucciniales order, and lack of a predicted function, were considered to select candidate secreted effector proteins (CSEPs)^21,22^. Recently, twenty *M. larici-populina* candidate effectors were shown to accumulate in multiple leaf cell compartments and target several protein complexes when expressed heterologously in *N. benthamiana*^22^. Of the CSEPs analyzed by Petre *et al*. (2015) and Germain *et al*. 2018^23^, Mlp124478 is the only one to localize to the nucleus and nucleolus both in *N. benthamiana* and *Arabidopsis*. *Mlp124478* is part of a gene family of nine members (CPG2811), which are specific to the order Pucciniales (Hacquard *et al*.^21^). *Mlp124478* expression is strongly induced during infection and reaches 50-fold induction at 96 h after infection. Given the kinetics of *M. larici-populina* infection, this corresponds to the biotrophic growth stage in mesophyll cells^24^. In addition, the CPG2811 group presents a signature of rapid evolution, a feature of pathogen effector families^21^. These different features observed for Mlp124478 prompted us to investigate its functional role more precisely.

Here, we confirmed the localization of Mlp124478 in epithelial cells of *A*. *thaliana*, we identify the sequence responsible for the nucleolar accumulation and investigate the effector cellular function *in planta*. Since the constitutive expression of *Mlp124478* in *A. thaliana* affects morphology and susceptibility to *H. arabidopsidis*, we used a transcriptomic approach to test whether the effector induces transcriptional reprogramming. Our results indicate that Mlp124478 nucleolar accumulation is dispensable for the effector to exert its virulence activity.

## Results

### Mlp124478 carries a putative nuclear localization signal and a putative DNA-binding domain

**Figure 1:**
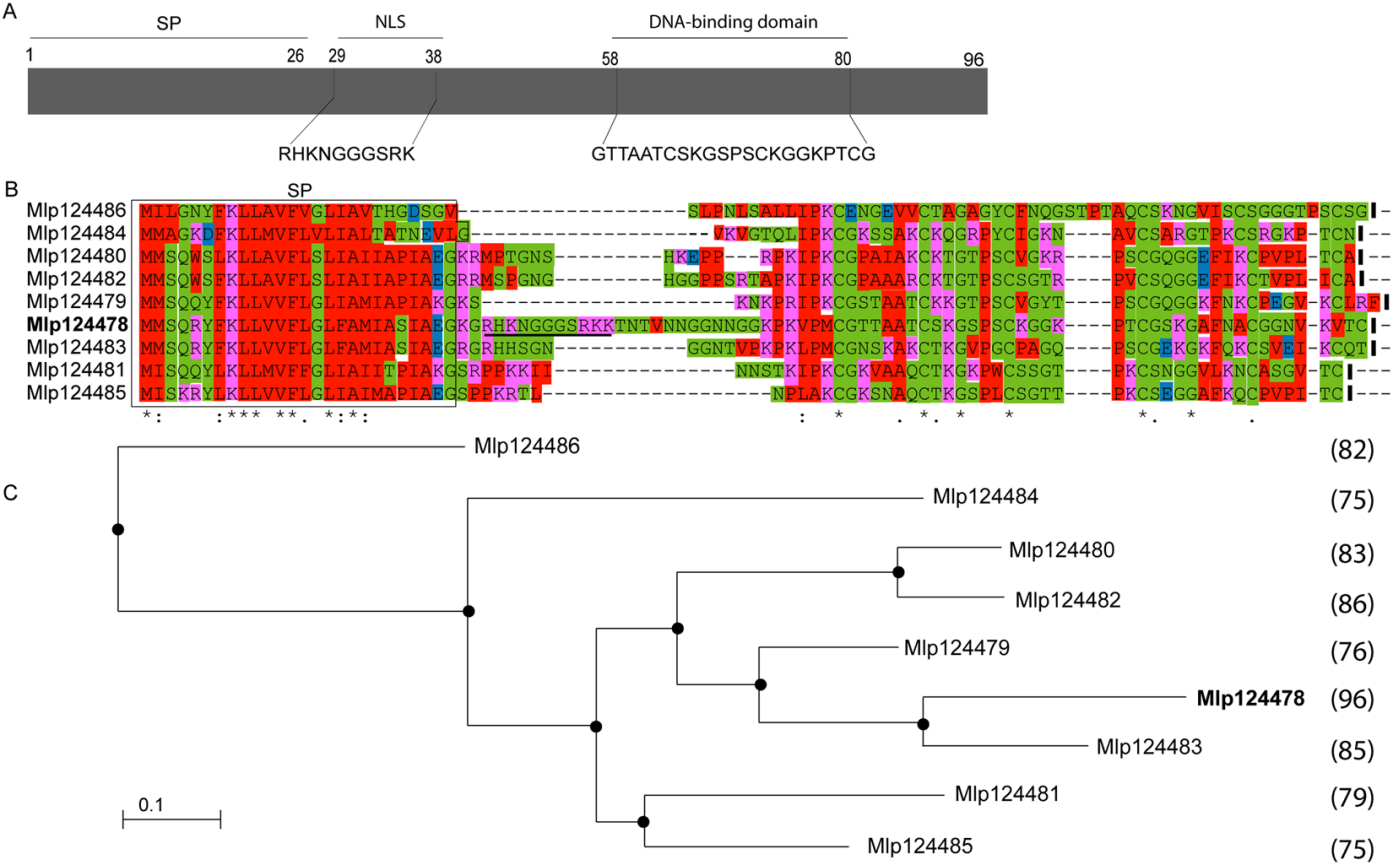
Sequence alignment and phylogenetic tree of the *M. larici-populina* CPG2811 SSP family. (**A**) Schematic representation of Mlp124478 protein topology, signal peptide (SP), nuclear localization sequence (NLS) and DNA-binding domain are shown. (**B**) Multiple sequence alignment of the nine members of the *M. larici-populina* CPG2811 SSP family. Predicted Signal peptides (SP) are boxed. Identical/highly conserved residues (*); semi conserved residues (:) and conserved residues (.) are marked. Predicted nuclear localization signal (NLS) is indicated by solid black underline. (**C**) Phylogenetic tree of the nine members of the CPG2811 gene family obtained with COBALT using Kimura distance value and neighbor joining tree method. Amino acid length is indicated in parenthesis.

Mlp124478 is part of the CPG2811 multigenic family, which is specific to rust fungi with nine members; each is composed of a predicted signal peptide followed by two exons encoding short peptides (75–96 amino acids) (Fig. 1A,B). Except for the six conserved cysteine residues, amino acid conservation is low in the family. Amino acid identity ranges from 28% to 60% between Mlp124478 and the other family members (Fig. 1C). Mlp124478 is the only member of the CPG2811 family that exhibits a putative nuclear localization signal (NLS) and a putative DNA-binding domain (amino acids 29–38 and 58 to 80, respectively, Fig. 1A). The infection specific expression of *Mlp124478* and its uniqueness among his family prompted us to investigate if it played a role *in planta* during pathogen growth.

### Mlp124478 affects *Arabidopsis* leaf shape and accumulates in the nucleus and the nucleolus of *A. thaliana* cells

**Figure 2:**
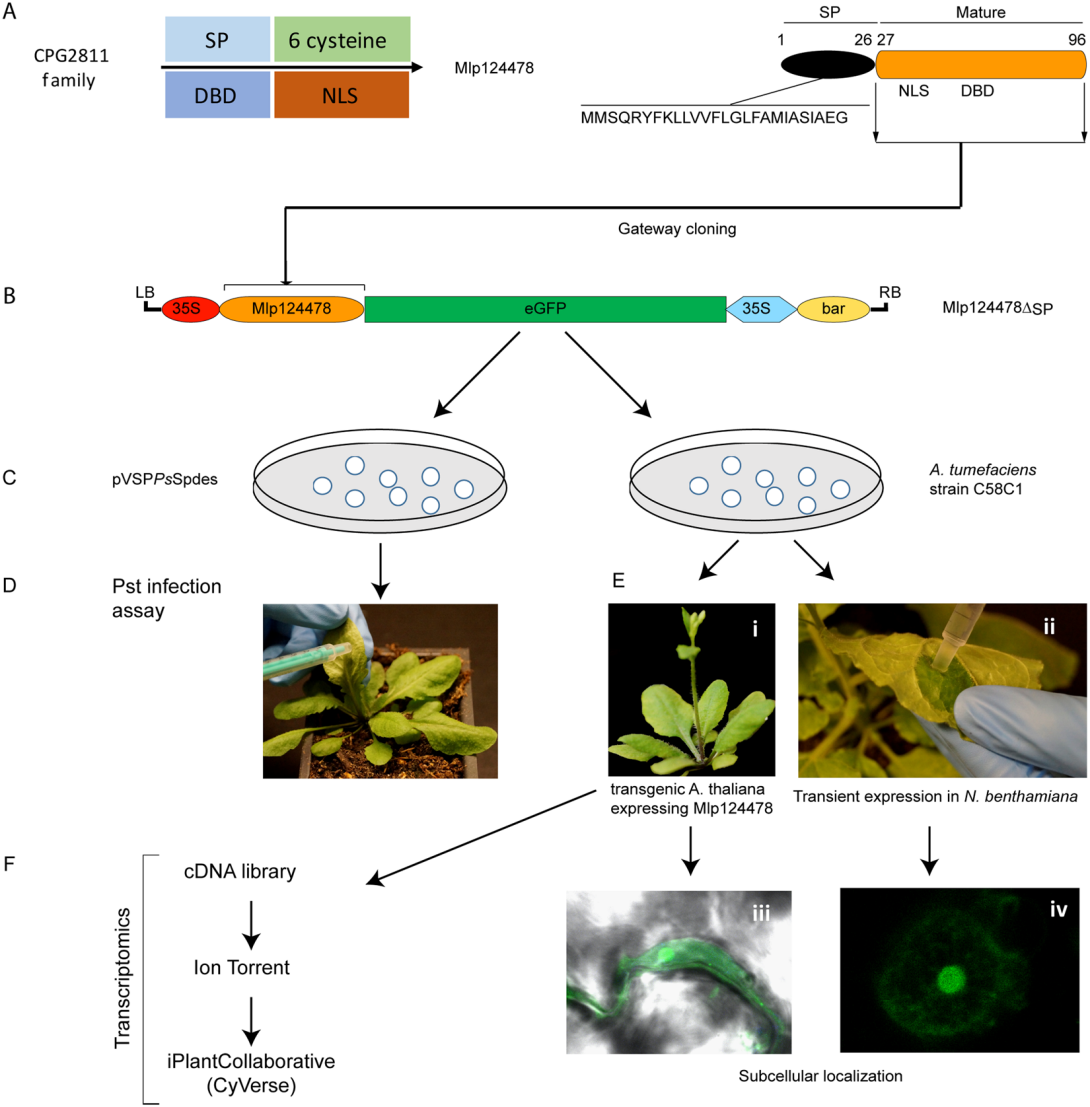
Overview of functional approaches applied to Mlp124478. (**A**) Mlp124478 was mined from CPG2811 family and has a signal peptide (SP), a putative nuclear localization signal (NLS) and a putative DNA-binding domain (DBD). (**B)** The mature coding sequences of Mlp124478 was cloned in frame with the green fluorescent protein (GFP). (**C**) Mlp124478 was recombined into pVSPPsSpdes vector for Pst infection assay (effector delivery) and pB7FWG2.0 was then inserted into A. tumefaciens strain C58C1. (**D**) Pst expressing Mlp124478 was syringe infiltrated into the abaxial side of the leaves of Arabidopsis thaliana. (**E**) A. tumefaciens strain C58C1 expressing Mlp124478 was used to develop stable transgenic A. thaliana plants expressing Mlp124478 and perform transient expression, both were viewed by confocal microscopy (**F**) Transcriptomic study was performed with cDNA library preparation from the RNA extracted from the transgenic A. thaliana expressing Mlp124478 and control.

**Figure 3:**
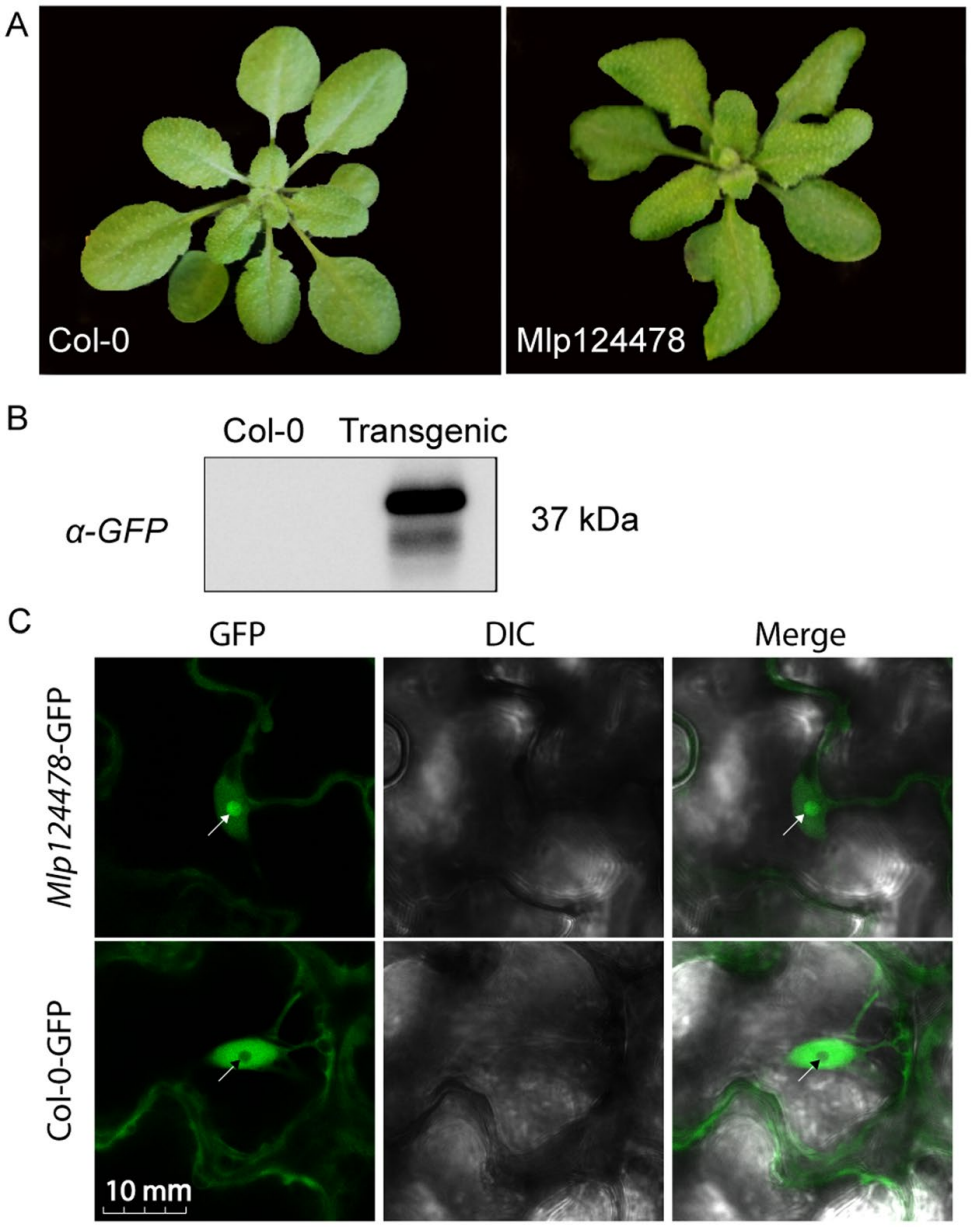
Mlp124478 carries a putative nuclear localization signal and a putative DNA-binding domain. (**A**) Morphology of 4-week-old soil grown *A. thaliana* Col-0 and stable transgenic plant expressing *Mlp124478* grown at 22 °C under 14 h/10 h photoperiod in growth chamber. (**B**) Immunodetection of GFP protein in Col-0 and stable transgenic seedlings from 12 days old plantlets. (**C**) Live cell imaging using confocal microscope of epidermal cells of 4-days-old *A. thaliana* stable transgenic *Mlp124478-GFP* plantlets. GFP in the Col-0 background was used as control. Left panel shows GFP, middle panel shows DIC and right panel shows merge. Nucleoli are pointed with black or white arrowheads.

To evaluate the biological consequences of Mlp124478′s presence in plant cells, we used functional genomic assays as summarized in Fig. 2. We generated a stable transgenic *A. thaliana* line expressing the mature form of *Mlp124478* (i.e., without the signal peptide) fused to GFP under the control of a 35S promoter (pro35S::*Mlp124478-GFP*) in the Col-0 background (Fig. 2). Interestingly, the transgenic lines exhibited altered leaf morphology, characterized by waviness of leaf margins, while no curvature in the margins was evident in Col-0 plants (Fig. 3A). Anti-GFP immunoblotting for proteins extracted from Mlp124478-GFP and Col-0 lines revealed a band signal at the expected size of 37 kDa only in the transgenic line (Fig. 3B), indicating that the full length fusion accumulates in plant cells. Our results suggest that the constitutive *in planta* expression of the Mlp124478-GFP fusion alters leaf morphology.

To ascertain the subcellular localization of Mlp124478, we undertook confocal laser scanning microscopy of leaves from 4-day-old *A. thaliana* seedlings expressing *Mlp124478-GFP* fusion. We detected the GFP signal in the nucleolus, the nucleoplasm, and the cytosol of epithelial cells (Fig. 3C) similar to the localization observed in *N. benthamiana* by Petre *et al*. (2015b). In contrast, in control plants expressing GFP, the fluorescent signal accumulated only in the nucleoplasm and cytosol, and was excluded from the nucleolus (Fig. 3C). We conclude that Mlp124478-GFP mainly accumulates in the nucleolus and nucleoplasm of leaf cells, with a weak accumulation in the cytosol.

### Mlp124478 Nuclear Localization Signal (NLS) is required for nucleolar accumulation

**Figure 4:**
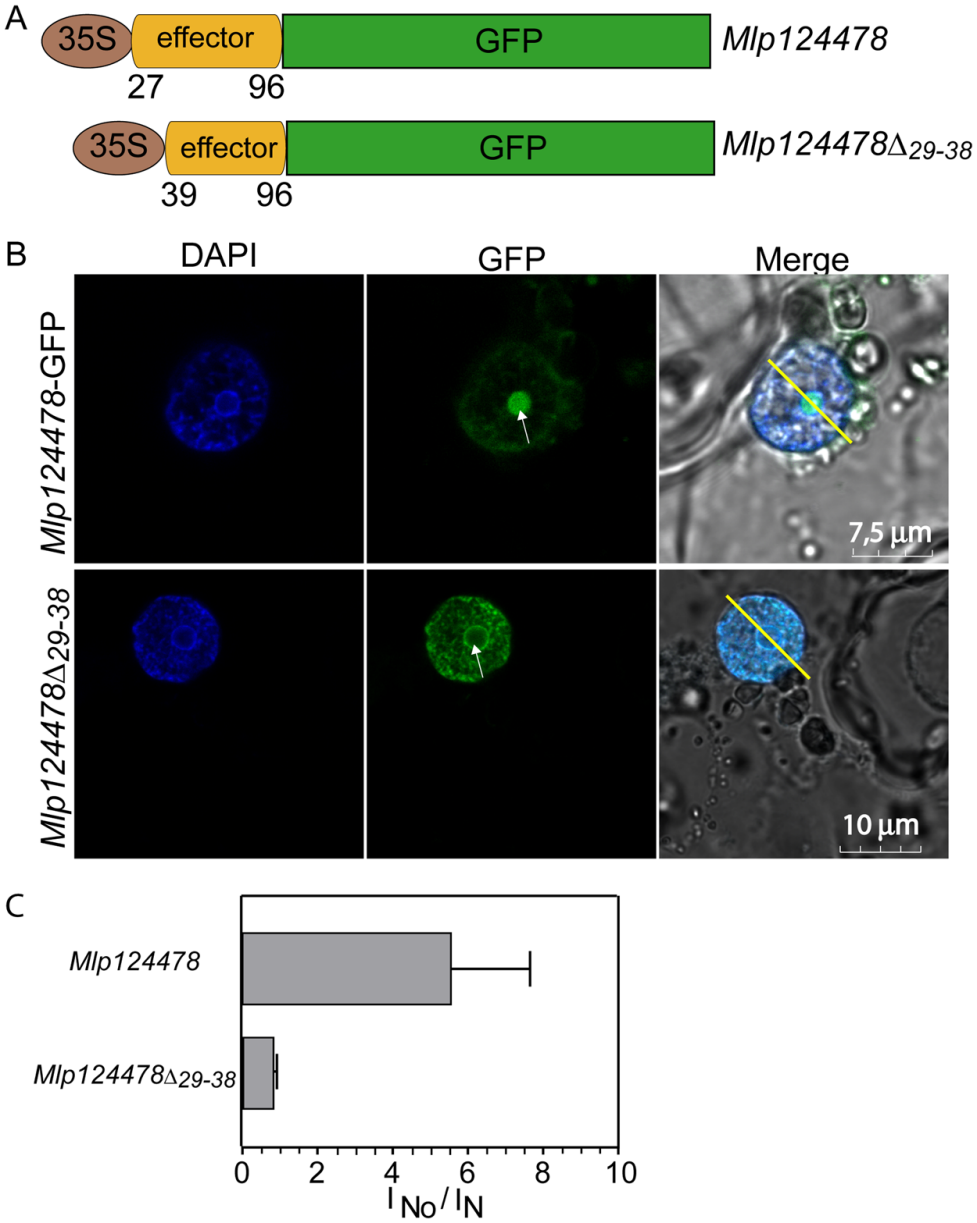
Mlp124478 Nuclear Localization Signal (NLS) is required for nucleolar accumulation. (**A**) Schematic representation of the constructs (Mlp124478 and Mlp124478∆_29–38_) used for transient expression. (**B**) Subcellular accumulation of Mlp124478-GFP and Mlp124478∆_29–38_-GFP in *N. benthamiana* epidermal cells at 4-days post-infiltration, the nucleus was stained by DAPI and epidermal cells were observed under the blue channel (left panel), green channel (middle panel) and merge of all channels (right panel). Arrowheads point the nucleolus. (**C**) Nuclear-nucleolar distribution of the fluorescent fusion proteins according to the fluorescence intensity ratios: nucleolar intensity (I_No_) divided by nuclear intensity (I_N_).

Mlp124478 carries a predicted NLS consisting of 10 amino acids within the N-terminal part of the mature form (Mlp124478_29–38_::RHKNGGGSRK) (Fig. 1). To assess whether the predicted NLS was required for nuclear localization, we designed a GFP tagged construct lacking the predicted NLS, hereafter named Mlp124478_Δ29–38_-GFP, and expressed it transiently in *N. benthamiana* leaf cells by agro-infiltration (Fig. 4A). Consistent with our observation in *A. thaliana* and from those of Petre *et al*. (2015), Mlp124478-GFP fusion accumulated in both the nucleus and nucleolus of *N. benthamiana* epithelial cells (Fig. 4B). However, Mlp124478_∆29–38_-GFP accumulated solely in the nucleus and cytosol, and its signal was excluded from the nucleolus (Fig. 4B). To quantify the changes in subcellular distribution, we generated intensity plots of the fluorescent signals, which showed decreased fluorescence in the nucleolus between the two Mlp124478 constructs (Fig. 4C and Supplementary Fig. 1). Mlp124478-GFP had a significantly higher nucleolar/nuclear ratio of 5.55 (SD = 1.55) compared to Mlp124478_∆29–38_ with a ratio of 0.8 (SD = 0.77) (Fig. 4C). Taken together, these results suggest that the predicted NLS of Mlp124478 also acts as a nucleolar localization signal.

### Mlp124478-GFP and Mlp124478_∆29–38_-GFP increase *H. arabidopsidis* growth on *A. thaliana*

In order to assess whether Mlp124478 accumulation and localization in plant cell affects susceptibility to pathogen growth, we generated an additional transgenic line expressing Mlp124478_∆29–38_-GFP and conducted two different pathogen assays. Firstly, we infected the stable transgenic *A. thaliana* that constitutively express effectors with *H. arabidopsidis*. Secondly, we used a *P. syringae* effector-delivery system.

**Figure 5:**
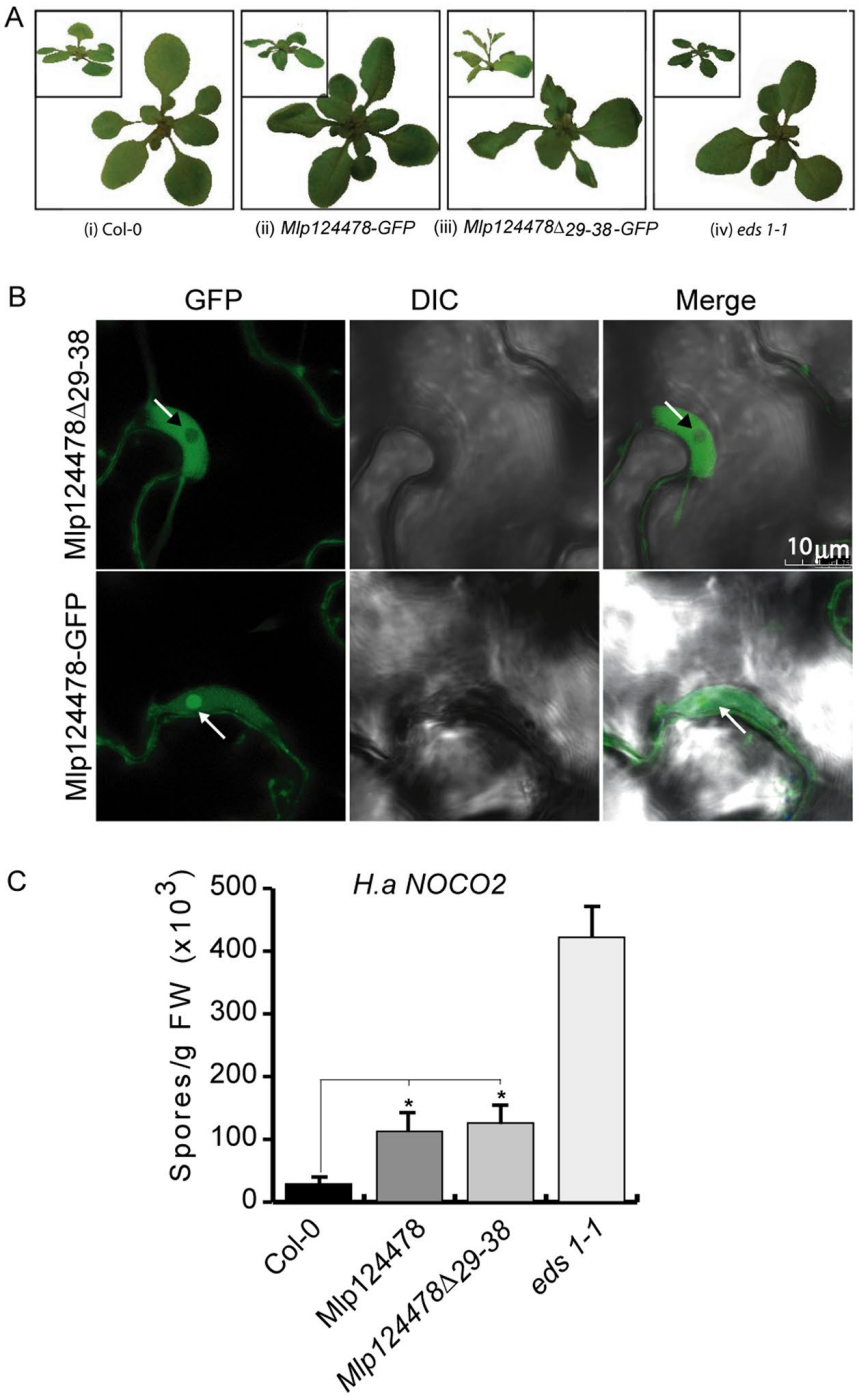
Mlp124478-GFP and Mlp124478_∆29–38_-GFP increase *H. arabidopsidis* growth on *A. thaliana*. (**A**) Col-0 plant showing normal leaves (i); wavy leaves phenotype observed in *Mlp124478-GFP* (ii); strongly enhanced leave waviness and early bolting in Mlp124478_∆29–38_-GFP (iii) morphology of *eds1-1* (iv). (**B**) Live cell imaging using confocal microscopy of epidermal cells of 4-days-old stable transgenic *Mlp124478*_*∆29-38*_*-GFP* and *Mlp124478-GFP* plantlets. Left panel displays GFP, middle panel shows DIC, right panel shows the merge. Nucleoli are pointed with arrows. Scale bar: 10 µm. (**C**) Four weeks old soil grown Col-0, stable transgenic *Mlp124478*, *Mlp124478*_*∆29*–*38*_*-GFP* and *eds1-1* plants were spray inoculated with *Hyaloperonospora arabidopsidis* Noco2 (50,000 conidiospores/mL) and the number of conidiospores were quantified at 7 days after inoculation. Statistical significance was evaluated using student’s *t* test. Asterisk denotes significant difference to Col-0, p < 0.0001 for Mlp124478 and p < 0.002 for *Mlp124478*_*∆29-38*_. Experiments were repeated three times with similar results.

Firstly, we observed that the wavy leaves phenotype observed in *Mlp124478-GFP* was strongly enhanced in Mlp124478_∆29–38_-GFP. It resulted in twisted and larger leaves (Fig. 5A-ii) and Mlp124478_∆29–38_-GFP plants also displayed early bolting (Fig. 5A-iii). We then confirmed that the effector localization in *A. thaliana* corroborated with the one observed in *N. benthamiana* and again observed a similar localization as before; that is Mlp124478-GFP effector accumulates in the nucleolus, nucleus and cytosol while the Mlp124478_∆29–38_-GFP is excluded from the nucleolus but still accumulate in the nucleoplasm and to a lesser extent in the cytoplasm (Fig. 5B). Secondly, we performed infection assays to evaluate the susceptibility of the transgenic plants. Seven days following *H. arabidopsidis* spores inoculation, we quantified the number of spores and counted 3.8 times more spores on Mlp124478-GFP than on Col-0, 4.3 times more spores on Mlp124478_∆29–38_-GFP than on Col-0 and 14.5 times more spores on *eds1-1* than on Col-0. We noted a significant increased susceptibility in *Mlp124478* and Mlp124478_∆29–38_-GFP transgenic plants compared to Col-0 (*P* < 0.0001), although not as strong as that encountered in *eds1-1* plants, used as positive control (Fig. 5C). These findings demonstrate that the nucleolar localization of *Mlp124478* is not necessary for the augmented plant susceptibility to *H. arabidopsidis*.

Experiments using the plant bacterial pathogen *Pst*DC3000∆CEL carrying *Mlp124478* or an empty vector (Supplementary Fig. 2A) and additional experimental assay using *Pst* in which the effector was expressed *in planta* did not demonstrate alteration of pathogen growth (Supplementary Fig. 2B). These results indicate that neither the full-length mature effector nor the truncated effector excluded from the nucleolus increased plant susceptibility to this bacterial pathogen. From this experiment set, we conclude that Mlp124478 enhances the growth of the filamentous pathogen *H. arabidopsidis* but not of the bacterial pathogen *P. syringae* in *A. thaliana*.

### The expression of Mlp124478 in plant cells alters *A. thaliana* transcriptome

**Figure 6:**
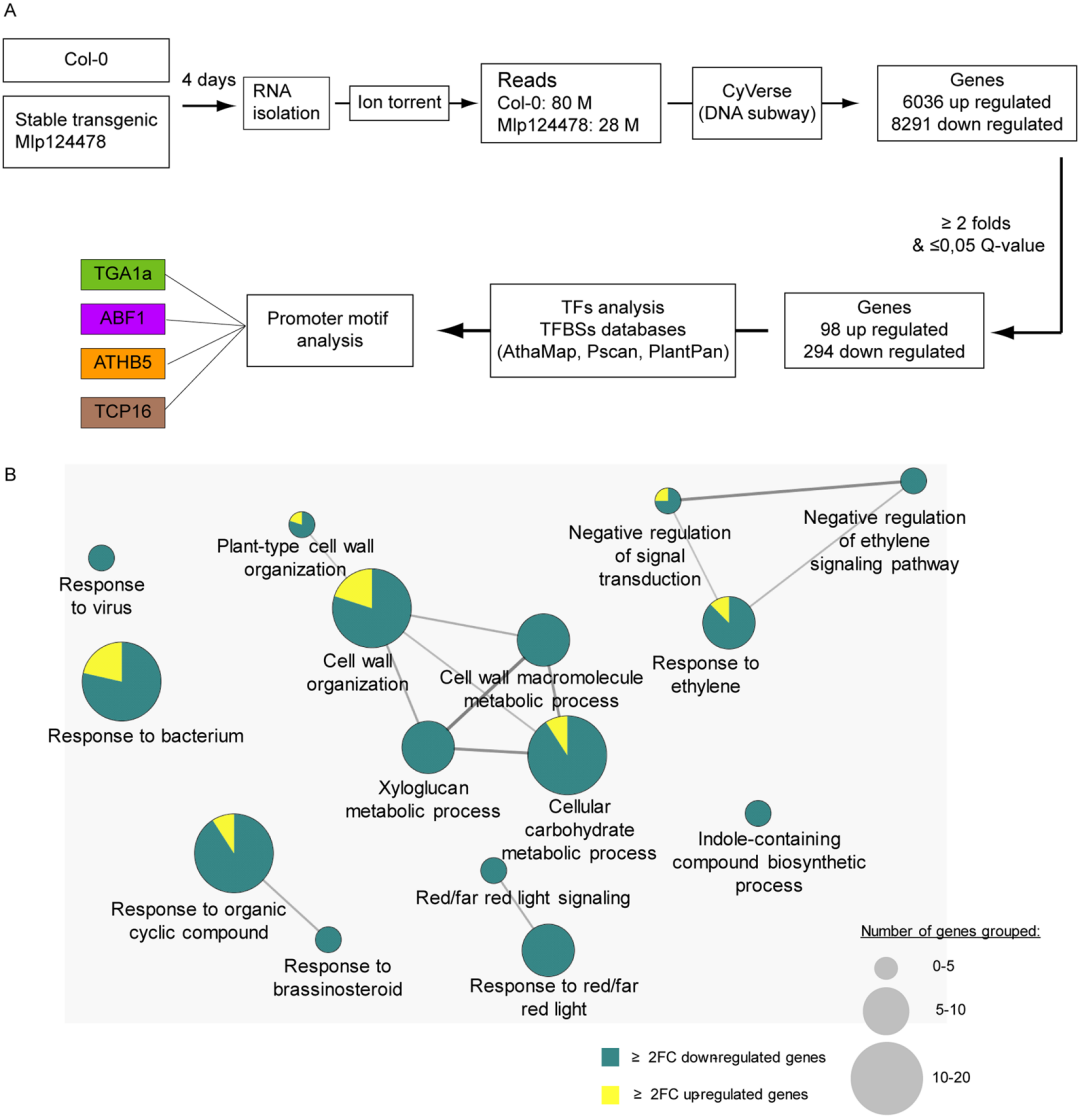
The expression of Mlp124478 in plant cells alters *A. thaliana* transcriptome. (**A**) Schematic illustration of transcriptomic work flow. RNA was isolated from 4-days old *A. thaliana* Mlp124478 stable transgenic and Col-0 plants and sequenced using Ion torrent. Transcripts were analyzed using iPlantCollaborative DNA subway and deregulated genes were considered for further analysis. (**B**) Go term enrichment analysis was performed with deregulated genes filtered with Q-value ≤ 0.05 and fold-change ≥ 2 using the Cytoscape software (version 3.1.1). Cytoscape was performed with the plug-in ClueGO and CluePedia to visualize functions enriched in the deregulated genes. The GO terms presented are significantly enriched in up-regulated and down-regulated genes with FDR ≤ 0.05 (Benjamini-Hochberg p-value correction) and revealed 15 GO terms belonging to 7 functional groups.

To better understand how Mlp124478 functions in plant cells, and since it alters plant morphology and susceptibility to pathogen, we investigated whether Mlp124478 alters gene expression in *A. thaliana*. We performed transcriptome profiling of 4-days-old *A. thaliana* Mlp124478 stable transgenic line and control plants expressing GFP. We identified 98 up- and 294 down-regulated genes, respectively (Fig. 6A, Supplementary Dataset). To test the robustness of the transcriptome data, we used qRT-PCR to assess the expression of 3 randomly selected up-regulated genes and 7 down-regulated genes. Transcriptome and qRT-PCR data correlated well, although quantitative differences were detected (Supplementary Fig. 3).

GO term enrichment analysis was applied to the deregulated genes to determine relevant biological processes. Seven functional groups (groups 0–6) of GO terms were significantly enriched among deregulated genes (Fig. 6B). The up-regulated genes with related GO terms are presented in (Fig. 6B). Among the 294 down-regulated genes and out of the 42 genes of the “cell wall organization”, 37 belong to the xyloglucan transglycolase XTH, XRT and EXT families. The defense-related transcription factors WRKY18, WRKY27, WRKY33, MYB51, the defense-related proteins NHL3, RPP8, YLS9, AZI1, CRK11 and the jasmonate pathway and regulation genes JAZ1, ASA1, ASB1 were down-regulated in the Mlp124478-GFP transgenic lines compared to the GFP transgenic plants. Other genes involved in diverse mechanisms were down-regulated such as the chitinase CHI, the brassinosteroid-related genes BAS1, BES1 PAR1, BEE1, the salicylic acid-related genes NPR3, the ethylene-related response genes ARGOS and ARGOS-like (ARL), EBF2, ERF6, ETR2, RTE1, the carbon metabolism-related genes EXO, the red/far red light signalization-related genes FAR1, GA2OX2, PAR1, PIF3, PKS4. The changes in Mlp124478-GFP *A. thaliana* transgenic line transcriptomes occured mostly by a down-regulation of the expression of genes involved in diverse functions, frequently related to defense response regulation.

**Figure 7:**
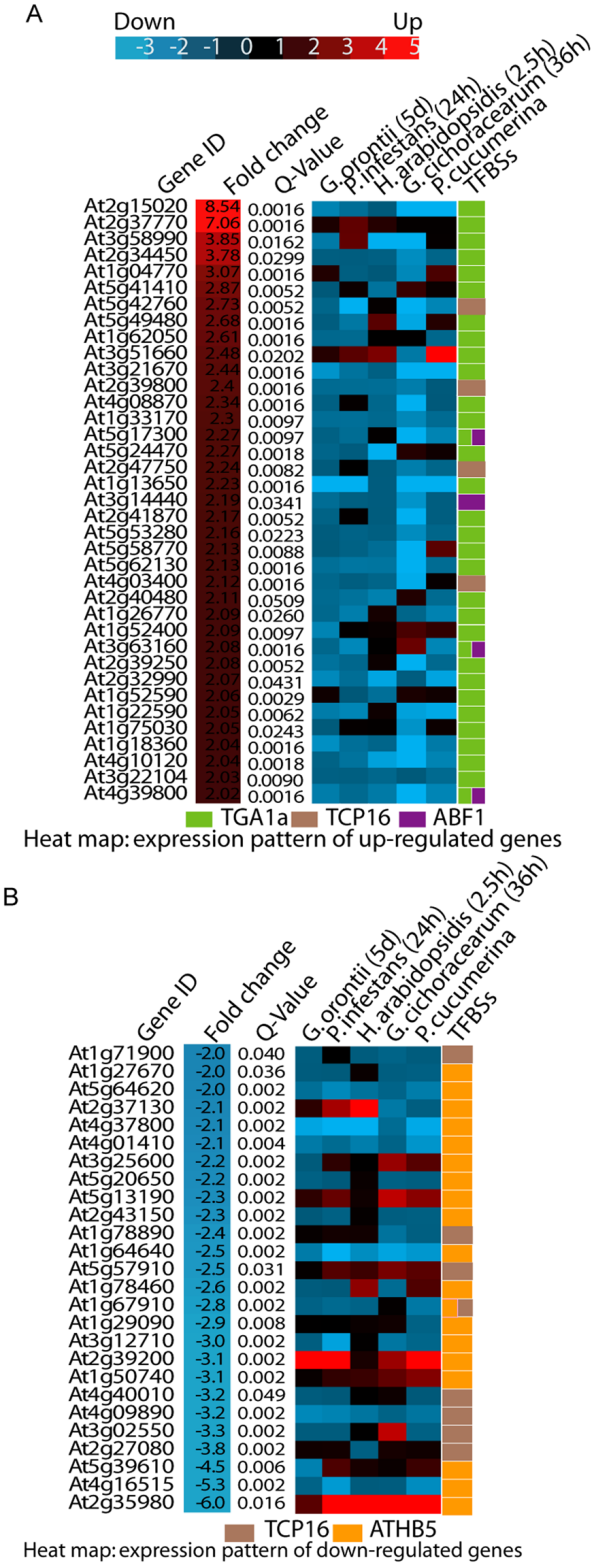
Regulation of gene expression level. Heat map of biotrophic pathogens response of genes in two groups: (**A**) upregulated genes and (**B**) down regulated genes. Genevestigator was used for differential expression analysis.

Next, we analyzed the gene expression profiles of up- and down-regulated genes during different biotic perturbations. We accessed Genevestigator towards this end. Expression levels in five different infection conditions (*Golovinomyces orontii*, *Phytophthora infestans*, *H. arabidopsidis*, *Golovinomyces cichoracerum*, *Plectosphaerella cucumerina*) were retrieved for all up- and down-regulated genes in the *A. thaliana* transgenic line overexpressing *Mlp124478* (Fig. 7A,B). Almost all up-regulated genes (92%) in our transcriptome analysis were down-regulated in response to these pathogens. Only one gene (At3g51660) also appeared up-regulated (maximum fold change of 2.5) in most conditions (Fig. 7A). Of the 30 down-regulated genes, 8 were up-regulated in almost all conditions surveyed (At2g37130, At3g25600, At5g13190, At5g57910, At2g39200, At1g50740, At5g39610, At2g35980; Fig. 7B). We further analyzed the identity of these genes. At2g37130 encodes a peroxidase, which was strongly up-regulated in response to fungal infection. At5g13190 encodes a plasma membrane protein regulating cell death. At2g39200 encodse *MILDEW RESISTANCE LOCUS O 12* (*AtMLO12)* whereas the product of the At2g35980 gene was very similar to *Arabidopsis NON-RACE-SPECIFIC DISEASE RESISTANCE 1* (*NDR1*). These results mirror those obtained with Cytoscape and further confirm that Mlp124478 rewires host transcription specifically to induce genes not normally expressed during infection against those five pathogens, whereas gene normally up-regulated in response to these pathogens are down-regulated upon expression of Mlp124478.

### Mlp124478 binds DNA

The localization of Mlp124478 *in planta*, the presence of a DNA-binding motif and alterations at the transcriptional, morphological, and immune levels prompted us to investigate whether Mlp124478 associates with DNA molecules. In a first time, we screened for Transcriptional Factor Binding Sites (TFBS) in the promoter sequences of all up- and down-regulated genes identified in our transcriptome analysis. We identified four different TFBS, which were very abundant among the up- (43 genes out of 98) and down-regulated (30 genes out of 294) genes (Supplementary Dataset). TFBS in the up-regulated gene set included ABF1 and TGA1a which belongs to the basic region/leucine zipper motif (bZIP) transcription factor (TF) family; and TCP16 belongs to the TCP (TEOSINTE BRANCHED 1, CYCLOIDEA and PROLIFERATING CELL NUCLEAR ANTIGEN FACTOR 1) TF family. The TFBS ATHB5 and TCP16 were also among the down-regulated genes. Thus, these TFBS were selected as candidate targets for Mlp124478-DNA interaction studies.

**Figure 8:**
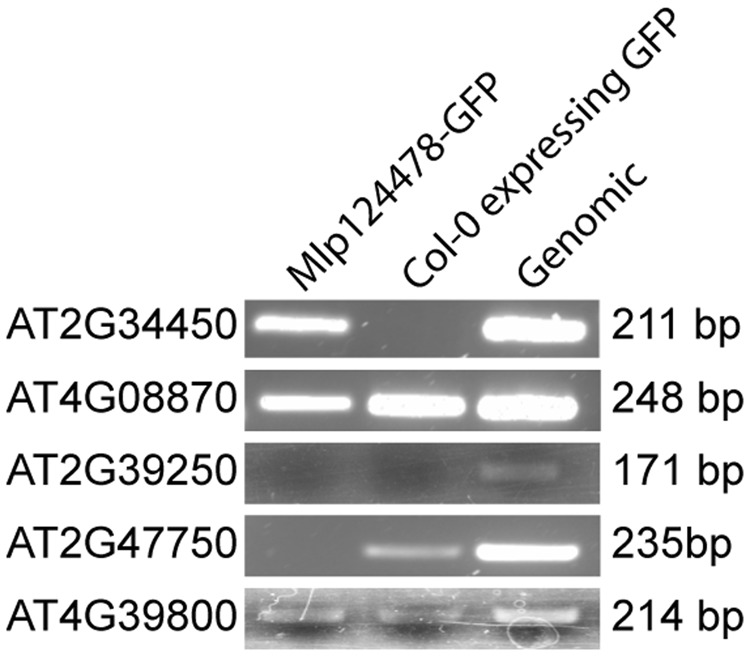
Mlp124478 binds DNA. Two-weeks-old plants tissues of Col-0 expressing GFP or stable transgenic *Mlp124478* were used for chromatin preparation using ChIP assay with antibody against GFP as described in the material and methods section and *A. thaliana* genomic DNA was used as a positive control. TGA1a associated site was PCR amplified with TGA1a specific primer pair. Expected bands (211 bp) was obtained from transgenic and *Arabidopsis* genomic DNA for TGA1a at the promoter region of AT2G34450 gene. Mlp124478-GFP indicates chromatin IP from that line. Col-0 indicates that chromatin was immunoprecipitated from Col-0 expressing GFP (negative control). Genomic Col-0 DNA, not immunoprecipitated served as a positive control. The other genes shown (At4g08870, At2g39250, At2g47750, At4g39800) are examples of non specific reaction. Col-0 expressing GFP DNA: negative control; *A. thaliana* genomic DNA: positive control.

In a second time, we performed a ChIP-PCR experiment. We cross-linked proteins and DNA using formaldehyde, and then immunoprecipitated (IP) Mlp124478-GFP fusion with anti-GFP beads from transgenic plants. We designed primer pairs that could amplify the promoter regions most abundant among de-regulated genes (TCP16, ATHB5, TGA1a and ABF1). One of the primer sets amplified DNA, revealing an interaction of Mlp124478 with the TGA1a-binding site of AT2G34450, one of the gene among the most strongly up-regulated genes in Mlp124478-expressing plants. We did not observe any band in the IP with Col-0 plant expressing GFP, which served as negative control, but a band was produced with *A. thaliana* Col-0 genomic DNA as positive control (Fig. 8) (four examples (At4g08870, At2g39250, At2g47750 and Atg39800) of non specific interaction are shown below At2g34450 (all interaction are shown in Supplementary Fig. 4)). AT2G34450 was up-regulated in the presence of Mlp124478 and showed down-regulation against biotrophic pathogens (Fig. 7A). We attempted EMSA with a synthetic peptide encompassing the DNA-binding domain of Mlp124478 and a double-stranded oligonucleotide displaying the consensus TGA1a sequence, but did not discern any interaction (Supplementary Fig. 5). This results confirms that Mlp124478 interacts with DNA but the exact binding domain, sufficient for interaction could not be identified.

Since poplars are natural the hosts of *M. larici-populina*, we searched for the sequence immunoprecipitated in the ChIP experiment in the *Populus trichocarpa* genome. The promoter of the gene model POPTR_0004s13630.1 exhibits 57% of identity with the AT2G34450 promoter. Both genes encode protein with a similar predicted function, with a conserved exon-intron structure (6 exons and 5 introns), and a TGA1a regulatory sequence in their promoter sequence (Supplementary Fig. 6), suggesting that DNA interaction in *Arabidopsis* could also occur in poplars.

## Discussion

Recently, several groups reported on the use of heterologous systems to investigate the function, localization, and interaction of effectors from biotrophic pathogens^6,7,22,25–29^. It has also been shown that many pathogens effectors target the nucleus and, in some cases, alter transcription^30–32^. Here, we undertook a functional genomics approach to study Mlp124478, a CSEP from the poplar leaf rust pathogen *M. larici-populina*. We conducted *in planta* pathogen assays, live-cell imaging, comparative transcriptomics, and protein-nucleic acid interaction to assess Mlp124478 function.

One of the major finding of our study is that Mlp124478 represses the expression of genes involved in defense response. The GO terms that were most significantly enriched were response to virus, response to bacterium, response to brassinosteroid, indole-containing compound biosynthetic process, cell wall organization, response to red or far red light signaling and negative regulation of ethylene-activated signaling pathway (Fig. 6B). Among the most down-regulated genes, several belonged to the defense-related transcription factors WRKY18, WRKY27, WRKY33, MYB51^33,34^, the defense-related proteins NHL3^35^, RPP8^36^, YLS9^37^, AZI1^38^, CRK11^39^ and the jasmonate pathway such as JAZ1^40^, ASA1, ASB1^41^, a chitinase which is involved in defense against fungi and salicylic acid-related genes NPR3^42^. Thus, the changes in the transcriptome of the transgenic line expressing *Mlp124478-GFP* occur mostly by the down-regulation of expression of genes involved in functions frequently related to defense response regulation.

The presence of a DNA-binding domain in Mlp124478 and the fact that we could confirm Mlp124478 interaction with DNA in a sequence-specific manner, besides alteration of the transcriptome through downregulation of defense related genes strongly suggest that it may alter gene expression to deceive plant immune systems. Recently, effectors from filamentous pathogens that bind DNA have been identified. CgEP1, a *Colletotrichum graminicola* effector with DNA-binding properties has been shown to enhance anthracnose development in maize^3^. Like Mlp124478, the oomycete effector PsCRN108 exhibits a putative DNA-binding domain, localizes to the nucleus and downregulate the expression of defense-related genes^43^. Several down-regulated genes found in our study corresponded to genes also reported recently highlighted in a transcriptomic analysis of *A. thaliana* responses during colonization by the two fungi *Colletotrichum tofieldiae* (symbiont) and *Colletotrichum incanum* (parasite). This study and our results share eight GO terms, with the exception that genes induced during the colonization by *C. incanum* are down regulated in Mlp124478 transgenic lines (Supplementary table 2). Hence, the expression of this single effector (Mlp124478) appears to bear broad transcriptional impact as it appears to counter the normal gene regulation described by Hacquard (2016) using a very similar analysis. However, since the EMSA assay could not confirm a direct protein-DNA interaction, the broad transcriptional changes caused by the presence of Mlp124478 could be caused by an indirect effect or interaction with a transcriptional regulator.

When Arabidopsis was exposed to a filamentous pathogen that possesses a mode of infection similar to rust fungi, we observed more susceptibility to pathogen growth, but susceptibility was not enhanced to infection by bacterial pathogen. This result indicates that this effector may target an immunity component specifically affected by pathogens with filamentous lifestyle. The morphology of the plants expressing *Mlp124478* or *Mlp124478*_*∆29*–*38*_ is altered, the plants show wavy leaves and *Mlp124478*_*∆29*–*38*_ displays early bolting. Altered phenotype has previously been associated to altered susceptibility to pathogen. For instance, the *scn1* mutant plants have been thoroughly described as having increased resistance to pathogen and accumulate elevated salicylic acid level^44^, however *snc1* plants have very short stature unlike the plants expressing *Mlp124478*. Although wavy leaf phenotype has been reported before it does not appear to be linked to plant susceptibility^45^.

Since the default GFP distribution in plant cells is nucleo-cytoplasmic, the localization of a GFP-tagged effector displaying nucleo-cytoplasmic distribution is considered non-informative. However, in the case of Mlp124478, the localization in nucleoli indicates that GFP is not masking the Mlp124478 localization sequence, thus localization is driven by the effector sequence. Nucleolus targeting has long been recognized as a hallmark of virus infection^46–48^, essentially to recruit nucleolar proteins and facilitate virus replication^48^, but has also been observed for other pathogens, including oomycete^49^ and bacteria^50^. While viral lifestyle easily explains the need to target the nucleolus, the reasons why a rust effector would do so are not as clear. Since the virulence activity of Mlp124478 does not require nucleolar accumulation, the accumulation of Mlp124478 in the nucleolus remains unexplained; however Mlp124478 could have additional function in the nucleolus which is undetected in our virulence assays.

Taken together, our results suggest that Mlp124478 likely manipulates plants by targeting DNA, remodeling transcription via DNA-binding, to suppress normal transcriptional responses to pathogens, and mislead the host into up-regulating the expression of genes unrelated to defense.

## Materials and Methods

### Plant material and growth conditions

*A. thaliana* and *N. benthamiana* plants were soil-grown in a growth chamber under a 14 h/10 h light/dark cycle with temperature set at 22 °C and relative humidity of 60%. The plants were grown in Petri dishes for the selection of single-insertion homozygous transgenic *Mlp124478* with ½ Murashige and Skoog medium containing 0.6% agar and 15 mg/ml Basta.

### Growth of *Pseudomonas syringae* pv. *tomato*, *H. arabidopsidis* Noco2 and infection assay

*Pseudomonas syringae* strain ∆CEL^51^ containing *Mlp124478* was grown overnight and infiltrated in the leaves of 4-weeks-old Col-0 and of transgenic *Mlp124478* plants at an optical density at 600 nm (OD_600_) of 0.001. *Pst* infections were produced by syringe infiltration of 4-weeks-old *Arabidopsis* plant leaves, and *H. arabidopsidis* Noco2 spray infections were performed as previously described^52^.

### Plasmid construction

Constructs were developed with Gateway cloning systems (Invitrogen, Life Technologies). The *Mlp124478* coding sequence without the signal peptide (lacking amino acids 1–27, hereafter referred to as *Mlp124478*) was ordered from GenScript in pUC57 in lyophilized form, and primer pairs (Supplementary Table 1, Primers Nos 1–3) were used to amplify the open reading frame (ORF) of *Mlp124478* from pUC57 by the polymerase chain reaction (PCR). Amplicons were then cloned into pDONR^TM^221 entry vector by Gateway BP recombination, followed by recombination with Gateway LR reaction either into pVSP*Ps*Spdes vector for *Pst* infection assay (effector delivery) or pB7FWG2.0 vector^53^ to express C-terminal green fluorescent protein (GFP)-tagged *Mlp124478* fusion *in planta*. pVSP*Ps*Spdes harbors the AvrRpm1 secretion signal^54^.

### Transient expression in *N. benthamiana* leaf cells

Solutions of *A. tumefaciens-*carrying recombinant plasmids were infiltrated into leaf pavement cells of 4-weeks-old *N. benthamiana* plants^55^. Briefly, *A. tumefaciens* AGL1-competent cells were transformed with pB7FWG2-containing *Mlp124478* and grown overnight in yeast extract peptone medium supplemented with spectinomycin (50 mg/L). The cells were precipitated by centrifugation at 300 *g* and adjusted to OD_600_ of 0.5 in infiltration buffer (10 mM MgCl_2_ and 150 µM acetosyringone). After 1 h, the agro-suspension was infiltrated into the abaxial side of leaves, and the plants were returned to the growth chamber. At 2 days post-infiltration (dpi), water-mounted slides of leaf tissue from agro-infected leaves were visualized by confocal microscopy.

### Confocal laser scanning microscopy

Leaves were observed under a Leica TCS SP8 confocal laser scanning microscopy (Leica Microsystems). Images were obtained with HC PL APO CS2 40X/1.40 oil immersion objective, and acquired sequentially to exclude excitation and emission crosstalk (when required). Leaves were immersed in water containing 0.2 µg/ml DAPI for 15 min for nuclei staining at room temperature. The samples were then observed at excitation/emission wavelengths of 405/444-477 nm and 488/503-521 nm for DAPI and eGFP, respectively. Images were annotated with LAS AF Lite software.

### Chromatin immunoprecipitation (ChIP)-polymerase chain reaction assay

ChIP assays were conducted, as described previously, with minor modifications^56^. Briefly, 300 mg of 2-weeks-old *A. thaliana Mlp124478* stable transgenics and Col-0 plants expressing GFP were collected in tubes containing 10 mL of phosphate-buffered saline (PBS), which were replaced by 10 mL of 1% formaldehyde to cross-link tissue under vacuum infiltration. To quench the cross-linker, 0.125 M glycine was added after removal of formaldehyde, followed by vacuuming, incubation for 5 min, and tissue-rinsing with 10 mL cold PBS. Cross-linked tissues were dried on paper towel for nuclei isolation. Sonicated chromatin was immunoprecipitated with 50 µL/mL anti-GFP microbeads (MACS, Miltenyi Biotec Inc.) and incubated for 2 h at 4 °C. The beads were placed in the µ-column, in the magnetic field of a µMACS separator, and washed twice. After reverse cross-linking of DNA-protein, ChIP samples underwent DNA purification according to a previously-described method^56^, followed by PCR amplification with specific primer pairs listed in Supplementary Table 1 (Primer Nos 4-38).

### Electrophoretic mobility shift assay (EMSA)

EMSA was undertaken, as described earlier^57^, with minor modifications. Unlabeled and digoxigenin (DIG)-labeled forward TGA1a oligonucleotides were ordered from Integrated DNA Technologies. Double-stranded (DS) oligonucleotides were annealed by heating 1 nmol of each oligonucleotide at 95 °C for 10 min, then slowly cooled down to 20 °C. DS oligonucleotides were diluted in TEN buffer (10 mM Tris-HCl, pH 8, 1 mM EDTA, pH 8, 100 mM NaCl) to a final concentration of 50 pmol/µL. Dot blotting was carried out by serial dilutions and spotted on positively-charged nylon membranes to test efficiency of the DIG-labeled probe. 3 pmol of probe was found to be efficient for detection with anti-DIG primary antibody. Gel shift reaction was performed with 3 pmol of DS oligonucleotides and 100 ng of synthetic peptide in binding buffer (100 mM HEPES, pH 7.6, 5 mM EDTA, 50 mM (NH_4_)_2_SO_4_, 5 mM DTT, 1% Tween 20 and 150 mM KCl). After binding reaction at 25 °C for 15 min, the samples were placed on ice for 15 min, and the mixtures were electrophoresed immediately through 0.25X TBE 20% polyacrylamide gel at 12.5 volts/cm. Bio-Rad semi-dry transfer cells were electroblotted on positively-charged nylon membranes at 25 volts for 10 min. DNA was then cross-linked to the membrane by baking at 80 °C for 40 min. For DIG detection, the membranes were blocked in TBS (50 mM Tris, 150 mM NaCl, 1% BSA), followed by 2 washes with TBS for 10 min and 1 wash with TBST (TBS and 1% Tween 20), then incubated overnight at 4 °C with anti-DIG monoclonal antibody diluted 1:1,000 in TBS with 1% BSA. The membranes were washed 4 times in TBS for 5 min and once in TBST. Finally, they were incubated with HRP-conjugated secondary antibody diluted 1:3,000 in TBST with 5% milk at room temperature for 45 min. The membranes were washed 4 times in TBS and once in TBST for 5 min. Bio-Rad’s Clarity Western ECL blotting substrate was then applied for detection. EMSA was performed at least 3 times with independent dilution of synthetic peptides and freshly-hybridized DIG probe.

### RNA extraction and transcriptome analysis

Total RNA was extracted from 4-days-old *A. thaliana Mlp124478* stable transgenics and from control plants expressing GFP with the RNeasy Plant Mini Kit (Qiagen, Inc.), according to the manufacturer’s specifications. The growth stage (Petri grown 4-days-old seedlings) was chosen to avoid variation due to growth chamber variation or micro-environmental variation, which would results in noise in the transcriptome analysis. Control and transgenic plants were extracted in triplicate. Eluted total RNA was quantified and sent to the Plateforme d’Analyses Génomiques of the Institut de Biologie Intégrative et des Systèmes (Université Laval, Quebec City, Canada) for library construction and sequencing with the Ion Torrent Technology. Differential expression was analyzed with green line workflow of the DNA subway in the iPlant collaborative pipeline (now CYVERSE) (Cold Spring Harbor Laboratory), including *A. thaliana*-Ensembl TAIR 10 as reference genome. Genes with a Q-value ≤ 0.05 and a fold-change ≥ 2 were considered as significantly differentially expressed and were further investigated for Gene Ontology (GO) enrichment. The Cytoscape software (version 3.1.1)^58^ with the plug-in ClueGO and CluePedia^59^ was used to visualize functions enriched among deregulated genes. The threshold for GO terms deregulation was set as FDR ≤ 0.05 (Benjamini-Hochberg p-value correction).

### qRT-PCR validation of the transcriptomic analysis

For qRT-PCR total RNA was extracted with the RNeasy Plant Mini Kit (Qiagen, Inc., Valencia, CA, USA) according to the manufacturer’s instructions. RNA quality was assessed by agarose gel electrophoresis and quantified by spectrophotometry. One μg of each sample was reverse transcribed into cDNA with the High Capacity cDNA Archive Kit (Life Technologies, Burlington, ON, Can). Quantitative RT-PCR (RT-qPCR) amplification was undertaken with a BioRad Detection system using SYBR Green PCR Master Mix (Bioline, London, U.K.). 100 ng cDNA template and 0.4 μM of each primer (listed in Supplementary Table 1) (were used in a final volume of 20 μl. The qRT-PCR thermal profile was: 95 °C for 2 min, 40 cycles of 95 °C for 5 s, 58 °C for 10 s, and 72 °C for 5 s. To analyze the quality of dissociation curves, the following program was added after 40 PCR cycles: 95 °C for 1 min, followed by constant temperature increases from 55 °C to 95 °C. Actin 1 served to normalize all RT-qPCR results. The expression levels of each gene were calculated according to the ∆∆Ct method^60^. Three technical replicates for each treatment were analyzed. Standard deviation was computed by the error propagation rule.

### Bioinformatics analyses

Clustal Omega (http://www.ebi.ac.uk/Tools/msa/clustalo/) was used to align sequences of the nine gene members of the CPG2811 SSP family, which were later manually annotated. Phylogenetic trees were generated by COBALT (http://www.ncbi.nlm.nih.gov/tools/cobalt/cobalt.cgi). SignalP 4.0 (http://www.cbs.dtu.dk/services/SignalP/) predicted signal peptides. NLStradamus (http://www.moseslab.csb.utoronto.ca/NLStradamus/) forecast nuclear-localizing signals. Transcription factor-binding sites (TFBS) were identified and analyzed with the AthaMap (http://www.athamap.de/search_gene.php)^61^, Pscan (http://159.149.160.88/pscan/)^62^ and PlantPan (http://plantpan2.itps.ncku.edu.tw/index.html)^63^ databases. Consensus TFBS sequences were retrieved from the Pscan database. Promoter sequences were obtained individually with TAIR’s SeqViewer (http://tairvm09.tacc.utexas.edu/servlets/sv), and TFBS-specific primers (Supplementary Table 1, Primer Nos 4-38) were designed with Primer3Plus (http://www.bioinformatics.nl/cgi-bin/primer3plus/primer3plus.cgi). Gene expression data under different biological conditions were retrieved from Genevestigator (https://genevestigator.com/gv/doc/intro_plant.jsp). Protein DNA-binding sites were predicted by MetaDBSite (http://projects.biotec.tu-dresden.de/metadbsite/)^64,65^.

## Electronic supplementary material

Supplementary Figures 1 to 6 and Supplementary Tables 1-2

Supplementary dataset
