# Supplementary material for: A rust fungal effector binds plant DNA and modulates transcription
Source: Sci Rep. 2018 Oct 3;8:14718. doi: 10.1038/s41598-018-32825-0 (PMC6170375; doi:10.1038/s41598-018-32825-0)

Legend of supplementary figures

Supplementary Fig. 1. Immunodetection of GFP protein in Col-0 and stable transgenic

Immunodetection of GFP protein in Col-0 and stable transgenic seedlings from 12 days old plantlets. The black square indicates the part that was cropped to show in Figure 3B.

Supplementary Fig. 2. Intensity plot of Mlp124478-GFP and Mlp124478Δ29-38-GFP.

Intensity plot presents fluorescence intensities between nucleus (N) and nucleolus (No). Intensity of BF, GFP and DAPI represented by gray, green and blue lines, respectively. Nu; nucleolar and N: nuclear.

Supplementary Fig. 3. *In planta*Mlp124478 presence increases *H. arabidopsidis* growth.

Quantification of *Pst*DC3000ΔCEL growth in Col-0 and stable transgenic *A. thaliana* expressing *Mlp124478*. Growth of bacteria was measured on days 0 and 3 (cfu: colony forming unit/mL inoculum). Statistical significance was evaluated using student's *t* test. Data were represented with the experiments repeated three times with similar results.B) Quantification of growth of *Pst*DC3000ΔCEL carrying or not *Mlp124478* in Col-0. Four weeks old plants were syringe infiltrated with bacteria at OD600=0.001. Growth of bacteria was measured on days 0 and 3 (cfu: colony forming unit/mL inoculum). Statistical significance was evaluated using student's *t* test. Data were represented with the experiments repeated three times with similar results.

Supplementary Fig. 4.Real-time PCR confirmation of the selected differentially expressed genes deregulated from transcriptome of A. thaliana expressing Mlp124478.

Relative transcript levels (log of fold change) of selected deregulated genes determined using real-time PCR, shown by black bar. Actin was used as the reference control. The fold change of deregulated genes from transcriptome data is shown by gray bars. The minus value means the gene is down-regulated; while the positive value means the up-regulation of genes.

Supplementary Fig. 5.Mlp124478 binds DNA (all non-specific results).

Two-weeks-old plants tissues of Col-0 expressing GFP, stable transgenic Mlp124478 were used for chromatin preparation using ChIP assay with antibody against GFP as described in the material and methods section and A. thaliana genomic DNA was used as a positive control. The genes shown in this supplementary figure all displayed non specific reaction. Col-0 expressing GFP DNA: negative control; A. thaliana genomic DNA: positive control. Full gels are shown.

Supplementary Fig. 6.Binding activity of TGA1a and DNA-binding domain of Mlp124478 by EMSA.

EMSA was carried out with DNA-binding domain specific synthetic peptide and digoxigenin labelled TGA1a probe. 10-fold excesses of oligonucleotides were added as competitors including wild type (TGA1a) and mutated (Mut-1 and Mut-2). The section cropped for the figure is identified by the black square.

Supplementary Fig. 7.Complete exon and intron structure with TFBSs at the upstream of gene.

Exons and introns structure of AT2G34450 and Ptp.5659.1S1_at.

Supplementary Fig. 1.

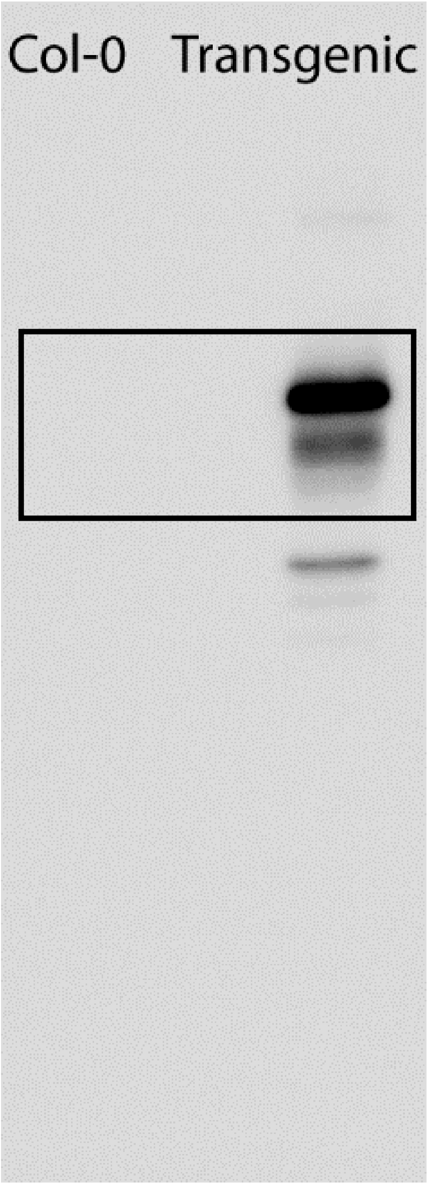

Supplementary Fig. 2

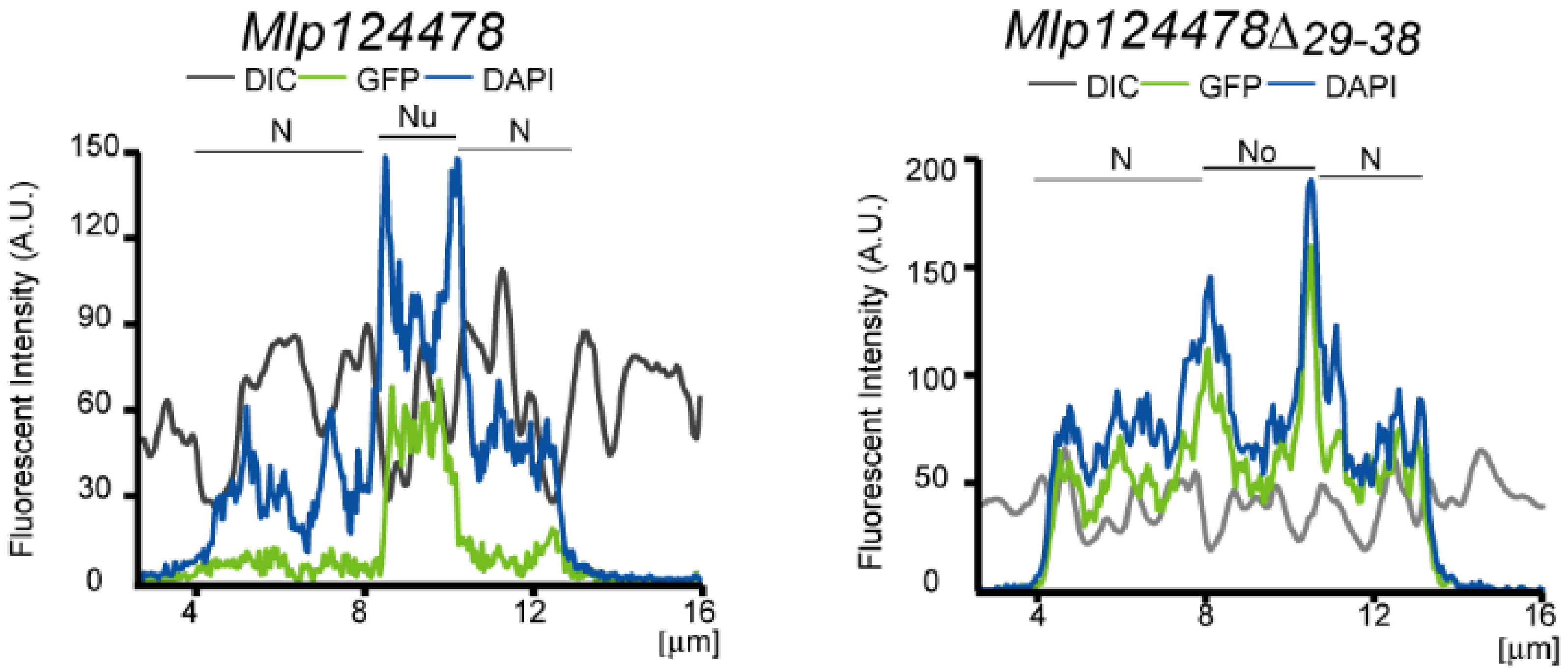

Supplementary Fig. 3.

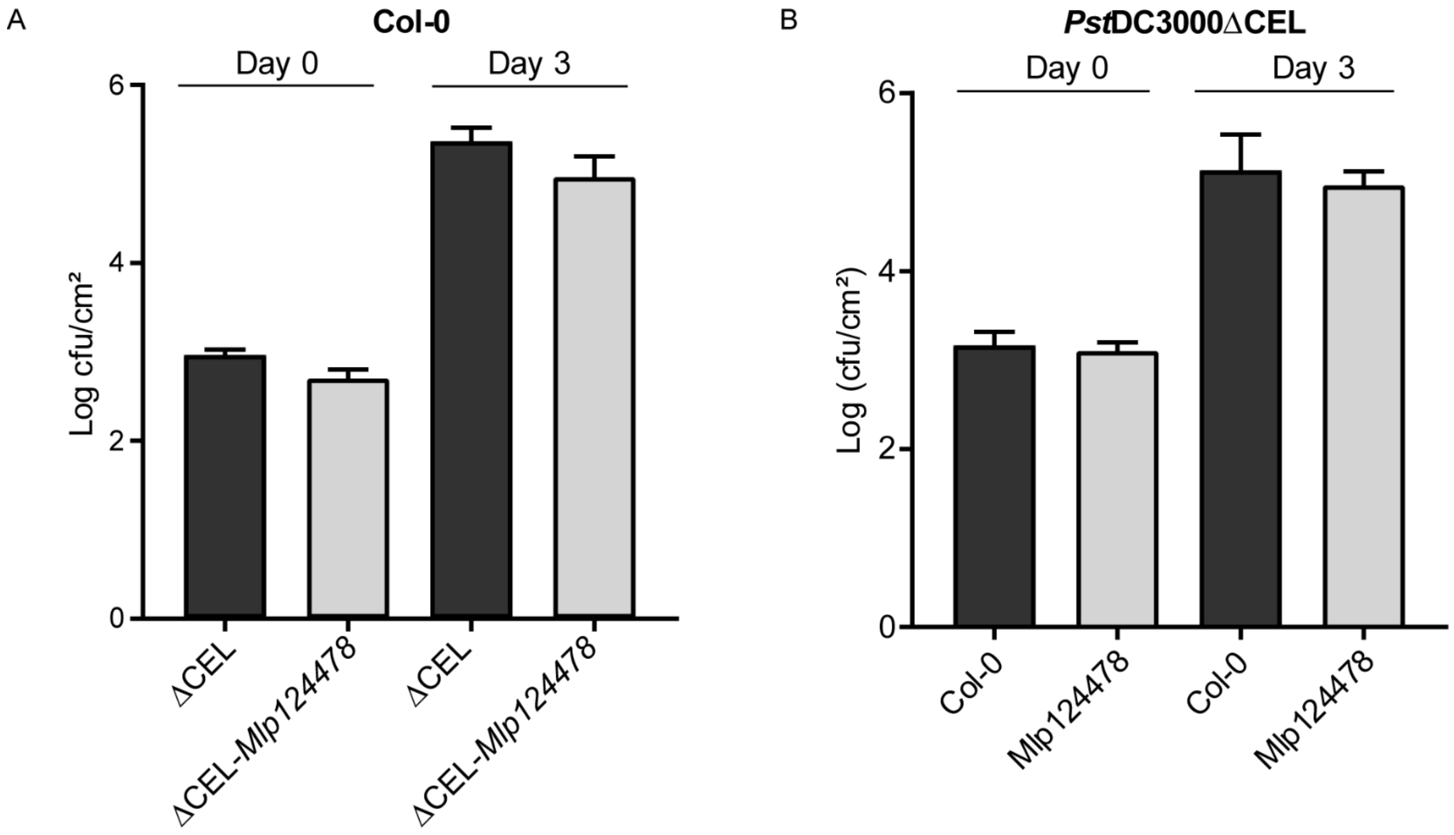

Supplementary Fig. 4

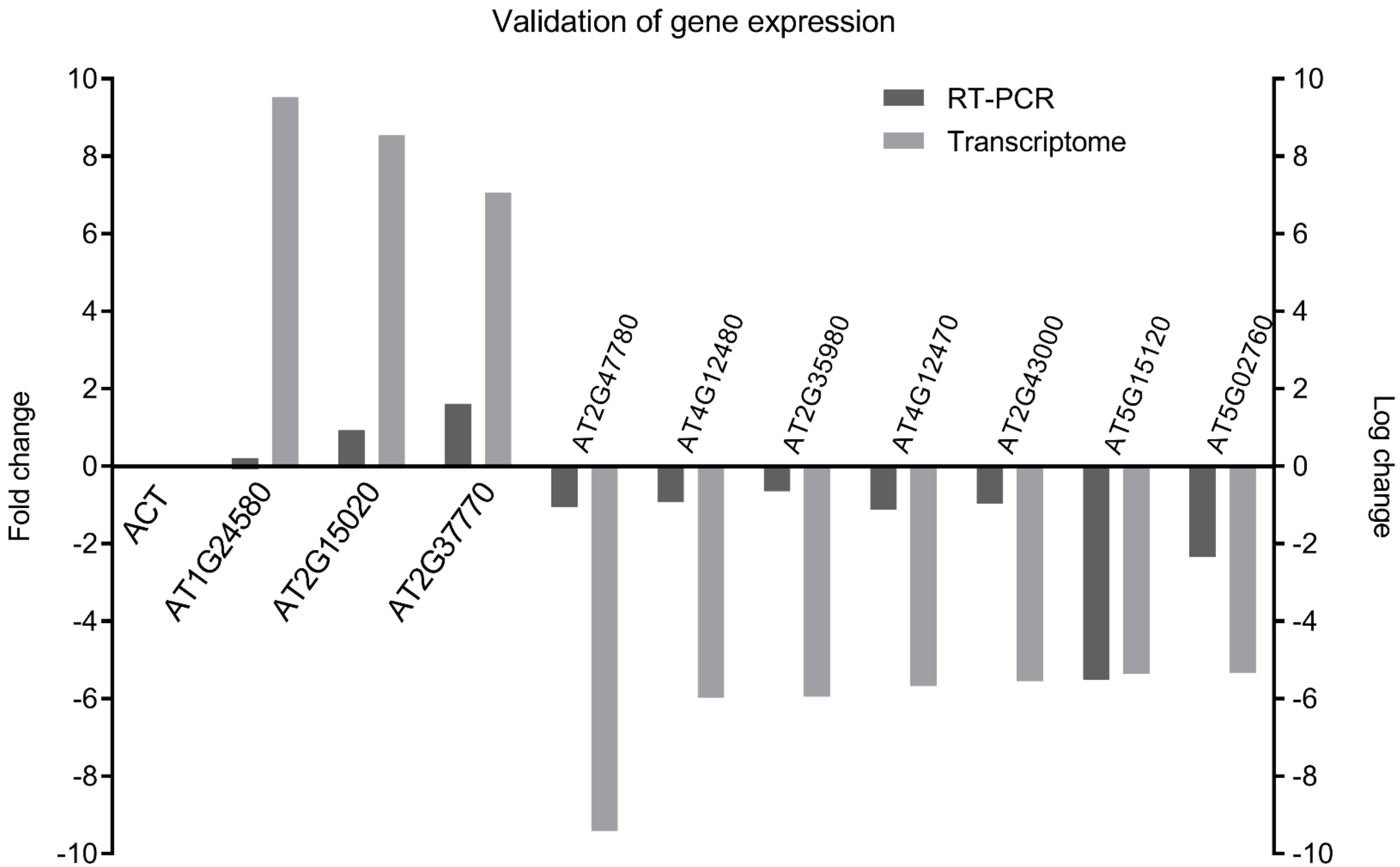

Supplementary Fig. 5

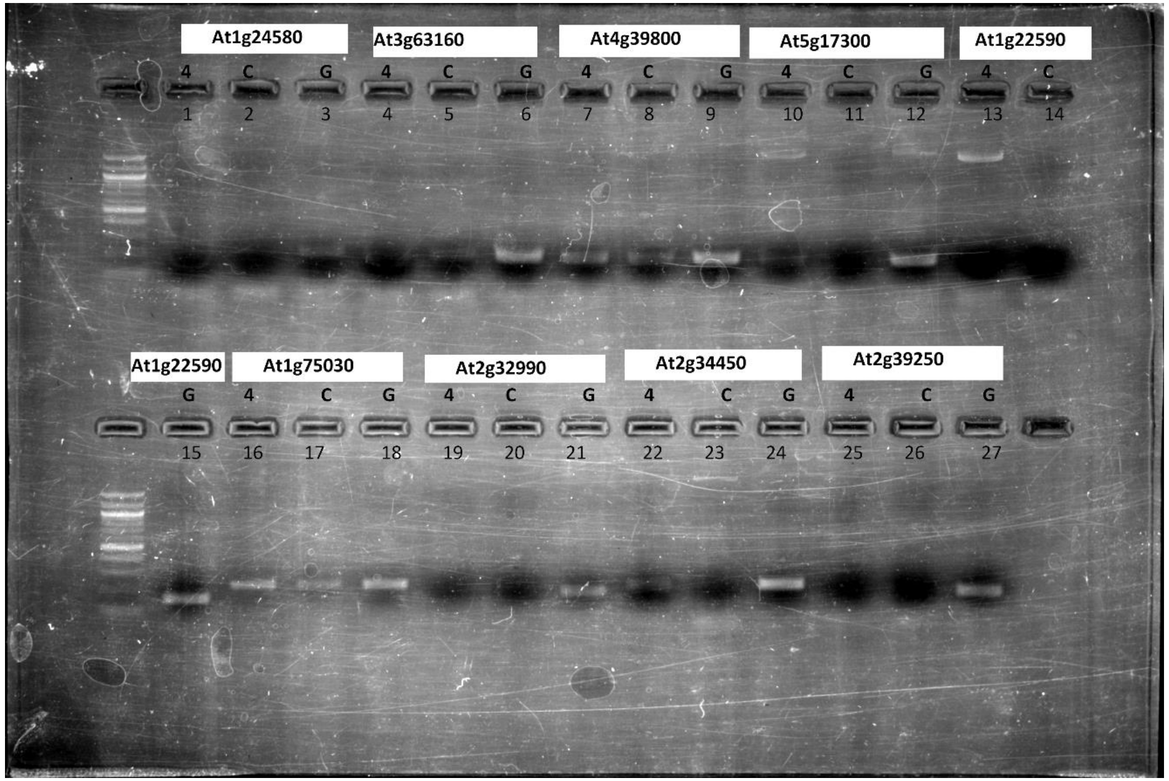

Supplementary Fig. 5 continued

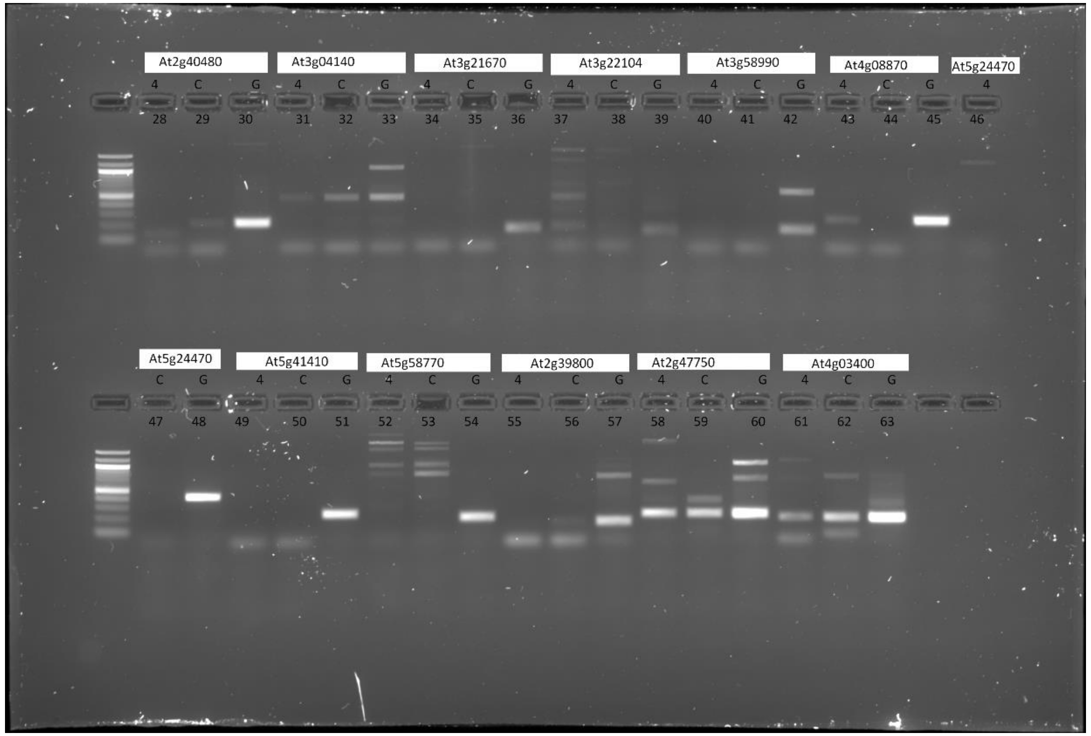

Supplementary Fig. 5 continued

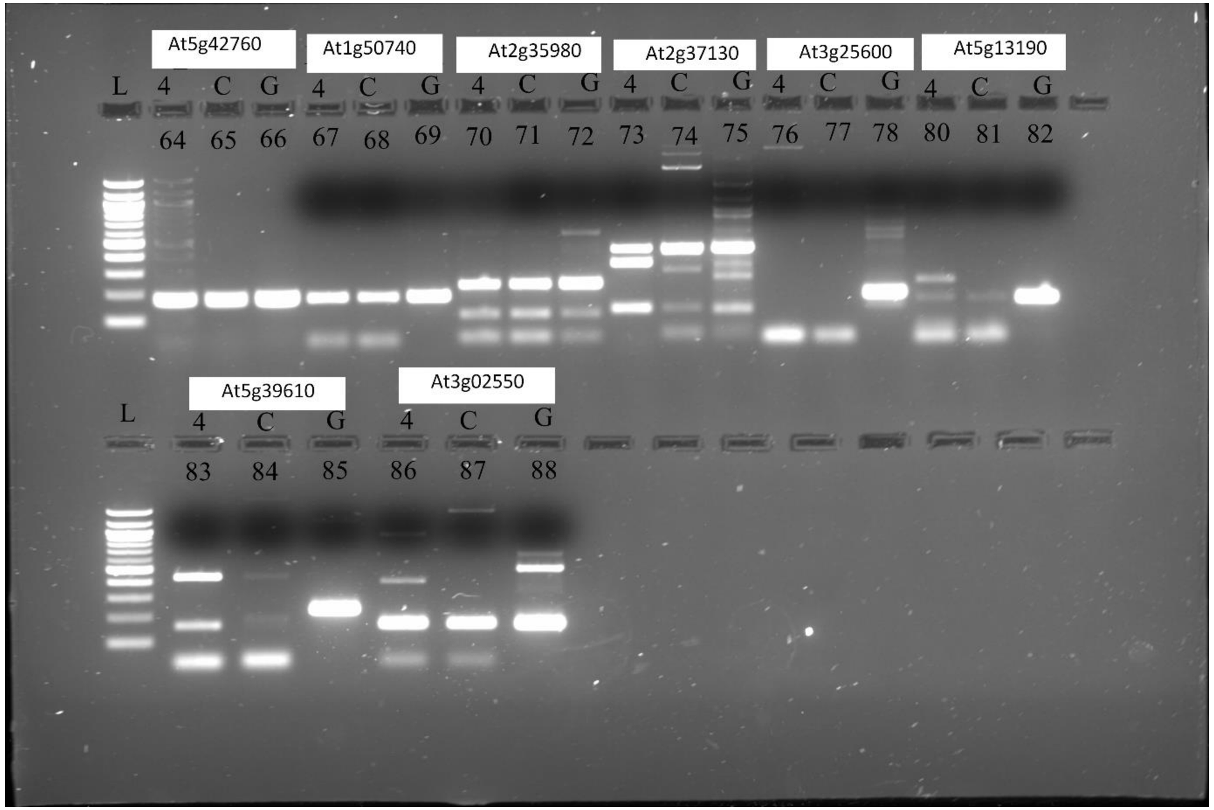

Supplementary Fig. 5 continued

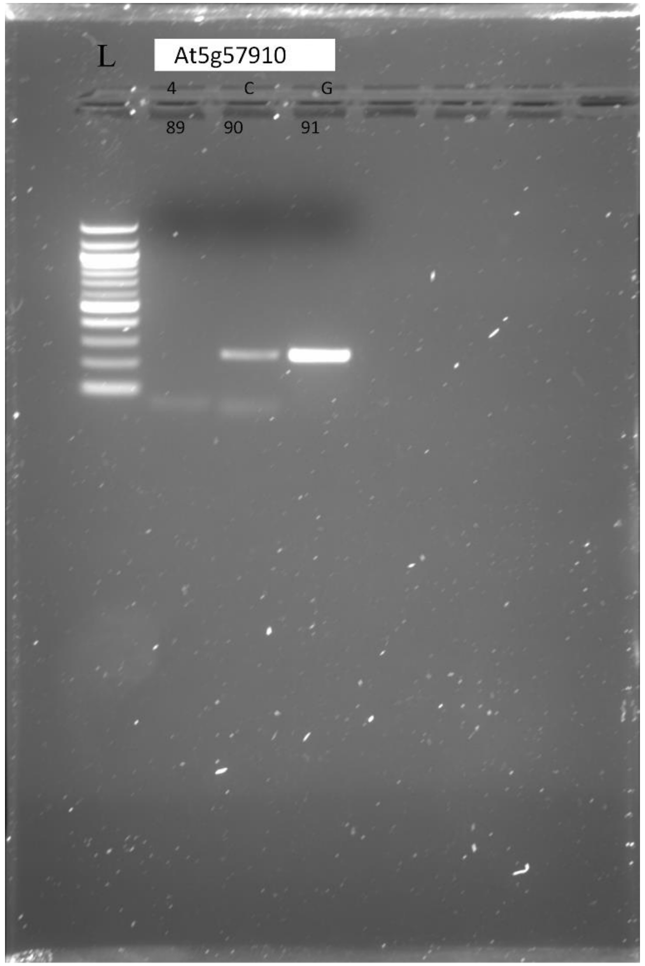

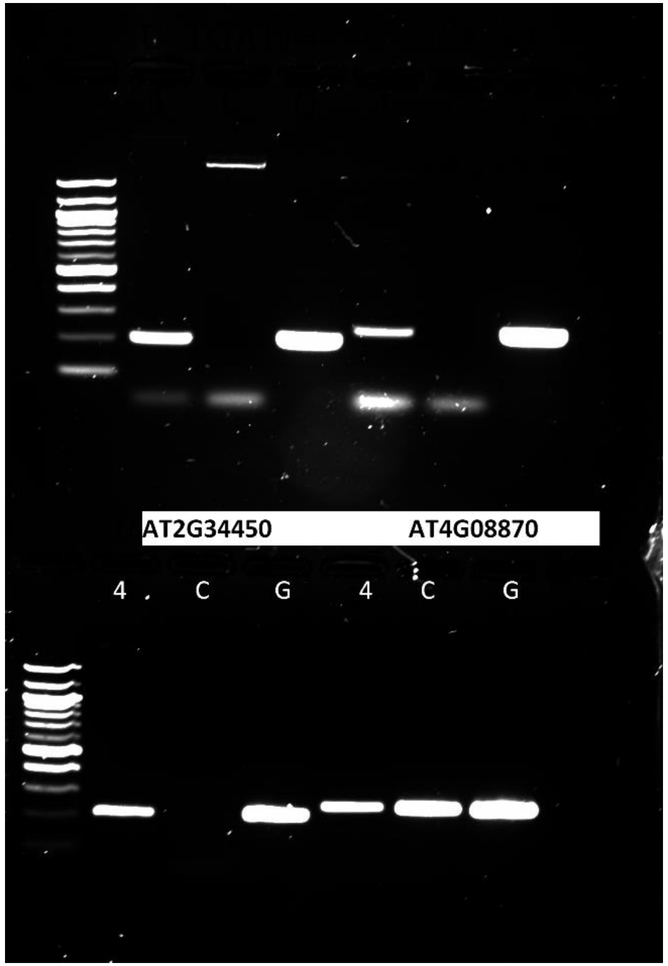

Supplementary Fig. 6

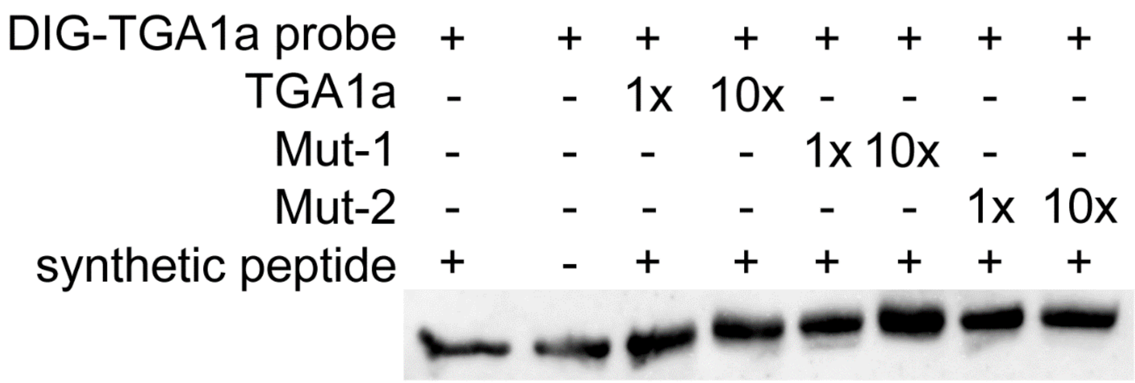

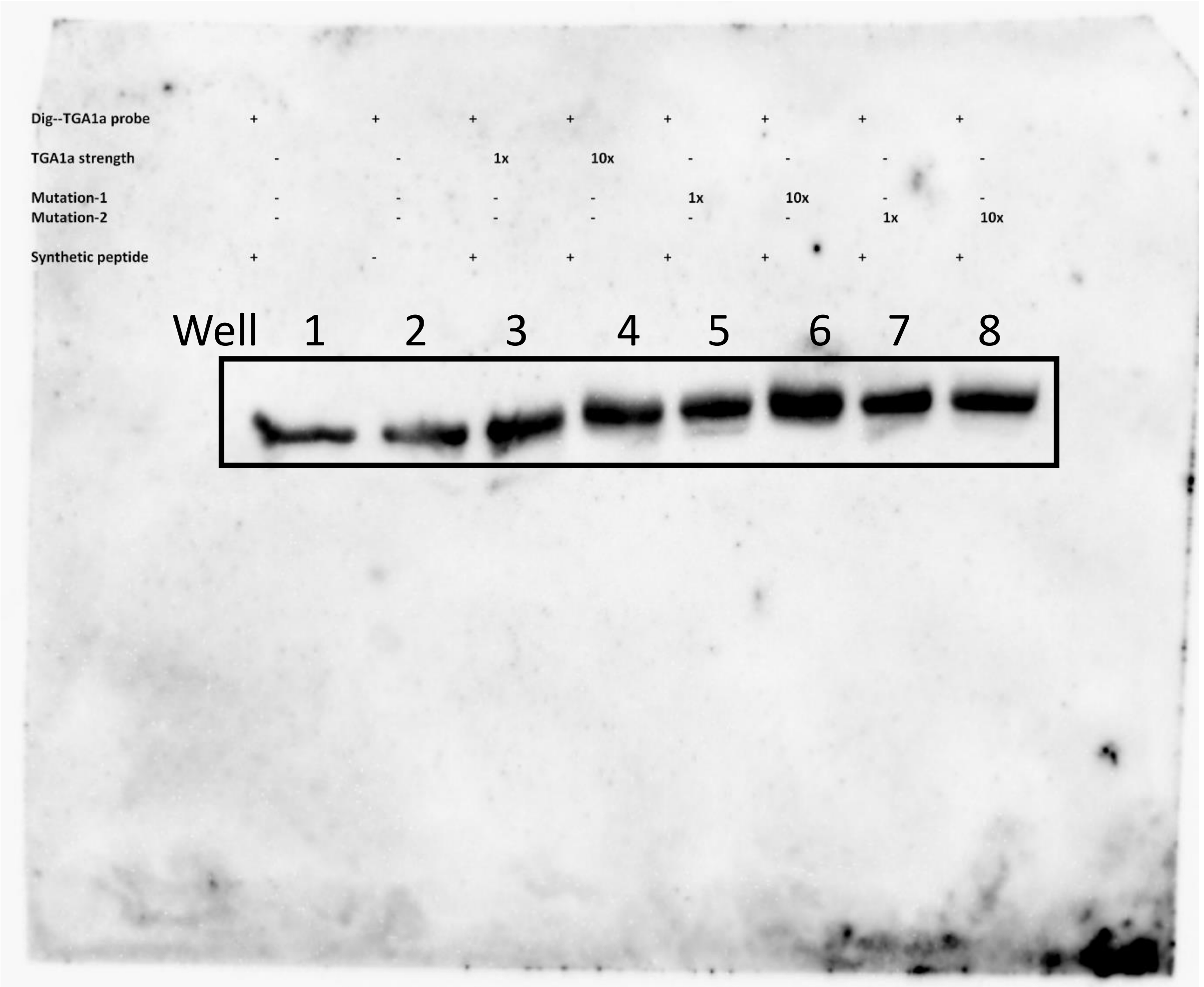

Supplementary Fig. 7

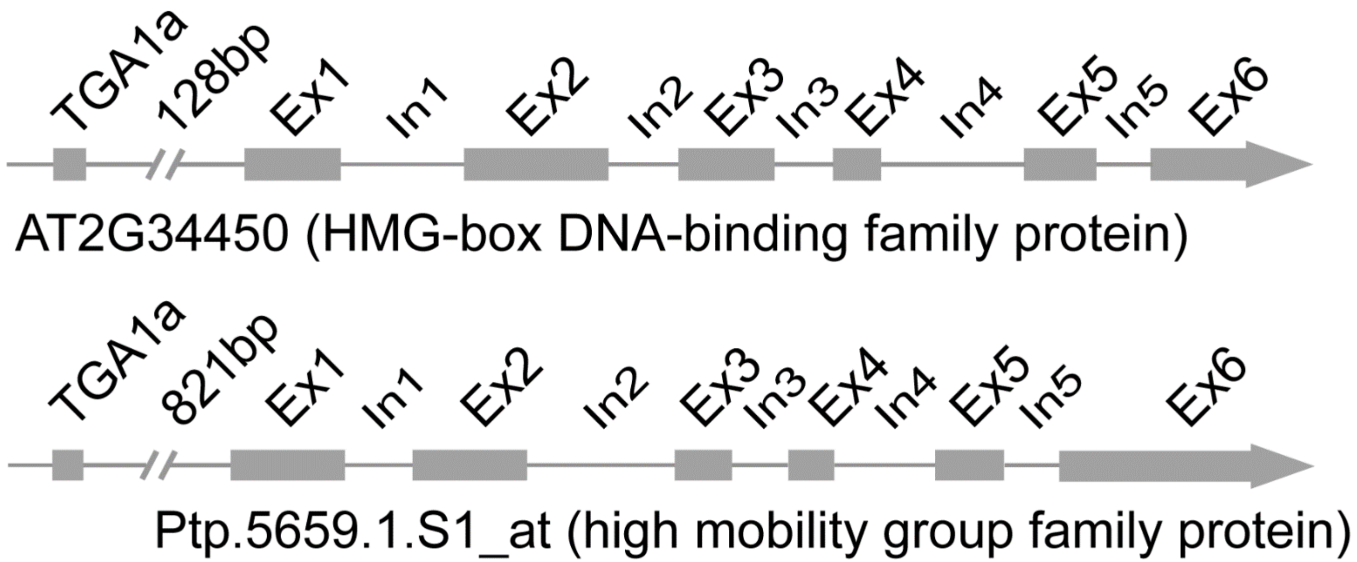

Supplement: Supplementary file 1 — Supplementary Figures 1 to 6 and Supplementary Tables 1-2 [file 41598_2018_32825_MOESM1_ESM.pdf]
